# Identification of biomarkers associated with diagnosis of acute lung injury based on bioinformatics and machine learning

**DOI:** 10.1097/MD.0000000000034840

**Published:** 2023-08-18

**Authors:** Hekun Jing, Xiaorui Chen, Daoxin Wang

**Affiliations:** a Department of Respiratory and Critical Care of the Second Affiliated Hospital of Chongqing Medical University, Chongqing, China.

**Keywords:** acute lung injury, bioinformatics analysis, biomarker

## Abstract

**Background:**

Acute lung injury (ALI) is an acute inflammatory disease characterized by excess production of inflammatory factors in lung tissue and has a high mortality. This research was designed for the identification of novel diagnostic biomarkers for ALI and analyzing the possible association between critical genes and infiltrated immune cells.

**Methods:**

The study used 2 datasets (GSE2411 and GSE18341) to identify differentially expressed genes (DEGs) between 2 groups. Then we performed Gene Ontology and Kyoto Encyclopedia of Genes and Genomes analyses to identify the functions of these DEGs. The study also used SVM-recursive feature elimination analysis and least absolute shrinkage and selection operator regression model to screen possible markers. The study further analyzed immune cell infiltration via CIBERSORT. Gene Set Enrichment Analysis was used to explore the molecular mechanism of the critical genes.

**Results:**

DEGs were identified between 2 groups. In total, 690 DEGs were obtained: 527 genes were upregulated and 163 genes were downregulated. We identified PDZK1IP1, CCKAR, and CXCL2 as critical genes. And we then found that these critical genes correlated with Mast Cells, Neutrophil Cells, M1 Macrophage, dendritic cell Actived, Eosinophil Cells, B Cells Naive, Mast Cells, and dendritic cell Immature. Furthermore, we investigated the specific signaling pathways involved in key genes and derived some potential molecular mechanisms by which key genes affect disease progression by use of Gene Set Enrichment Analysis. Moreover, we predict transcription factors. Also, we obtained critical gene-related microRNAs through the targetscan database, and visualized the microRNA network of the genes.

**Conclusion:**

Our findings might provide some novel clue for the exploration of novel markers for ALI diagnosis. The critical genes and their associations with immune infiltration may offer new insight into understanding ALI developments.

## 1. Introduction

Acute lung injury (ALI) and its more severe form, acute respiratory distress syndrome (ARDS), are acute inflammatory disease, characterized by excess production of inflammatory factors in lung tissue, and followed by non-cardiogenic dyspnea, severe hypoxemia, and pulmonary edema, thus confer substantial morbidity and mortality, and have no specific therapy.^[1–4]^ Its pathophysiological mechanism remains unclear after research of so many years. Therefore, the development of new diagnostic biomarkers reflecting lung injury is of great significance for the timely diagnosis and treatment of ALI.

Previous studies have shown that innate immunity is closely related to the prognosis of ALI.^[5]^ The immune system recognizes different pathogen-associated molecular patterns such as lipopolysaccharide (LPS) of bacteria and RNA of viruses through specific pattern recognition receptors, including toll-like receptors, Nod-like receptors, RIG-I-like helicases and the cytosolic DNA receptors. This recognition leads to the activation of the immune response.^[6]^ For example, cGAS can directly detect pathogens such as DNA viruses, retroviruses and bacteria. It binds and catalyzes the synthesis of 20’, 30’-cGAMP, thus triggering STING-dependent downstream signaling pathways that initiate the production and secretion of interferons to fight against pathogens.^[7]^

The recent high-flux genetic microarray analysis of specimens from sufferers and normal individuals enables us to investigate various diseases at diverse levels from somatic mutations and copy number variations to genomic expressions at the transcriptomic level, along with epigene variations. Recently, many specific genes have been discovered to participate in the progression of ALI. For instance, it was reported that Yu et al found that the protein level of RIPK3 was significantly increased in the plasma and bronchoalveolar lavage fluid of patients with ALI/ARDS. Knockdown of RIP3 in the murine model attenuated LPS-induced release of IL-1β, IL-6, and HMGB1, attenuated lung tissue damage and improved survival.^[8,9]^ These finding suggested the important roles of some function genes in ALI progression. However, the diagnostic value of many genes has not been investigated in ALI.

Here, we analyzed the ALI profiles that were downloaded from the Gene Expression Omnibus (GEO) database and performed the identification of differentially expressed genes (DEGs) between the ALI group and the normal group between ALI and healthy specimens. Then, we analyzed their diagnostic value in ALI based on machine learning. Overall, our findings provided novel critical genes involved in the progression of ALI.

## 2. Materials and methods

In the present study, ethical approval was unnecessary because all analytical data were derived from publicly available databases (https://www.ncbi.nlm.nih.gov/geo/).

### 2.1. Data download

The Series Matrix File of GSE2411 was downloaded from the NCBIGEO public database (https://www.ncbi.nlm.nih.gov/geo/) and annotated as GPL339. The expression profile data of 12 groups of samples were included, including 6 cases in the control group and 6 cases in the disease group. The Series Matrix File of GSE18341 was downloaded, and annotated as GPL1261. A total of 16 groups of sample expression profile data were included, including 8 cases in the control group and 8 cases in the disease group. The 2 datasets were merged, and the Combat algorithm was used for batch correction between chips. The limma package was used to identify differential genes between the control group and the disease group, and the screening conditions for differential genes were *P* value < .05 &| log FC|> 0.585.

### 2.2. Analysis of Gene Ontology and Kyoto Encyclopedia of Genes and Genome functions

In order to obtain the biological functions and signaling pathways involved in the occurrence and development of the disease, the Metascape database (https://www.metascape.org/) was used for annotation and visualization, and the differentially Gene Ontology (http://www.geneontology.org/) and Kyoto Encyclopedia of Genes and Genomes (https://www.kegg.jp/) pathway analysis. Min overlap ≥ 3 & *P* ≤ .01 was considered statistically significant.

### 2.3. Candidate diagnosis marker selection

We used least absolute shrinkage and selection operator (LASSO) regression and SVM algorithm for feature selection of diagnostic markers of disease. The LASSO algorithm uses the “glmnet” package. In addition, SVM-recursive feature elimination is a machine learning method based on support vector machines, which finds the best variables by deleting the feature vectors generated by SVM, and establishes support vector machine models through the “e1071” software package to further identify these biomarker pairs. diagnostic value of disease.

### 2.4. CIBERSORT analysis

The CIBERSORT method is a widely used method for the evaluation of immune cell types in the microenvironment. The method is based on the principle of support vector regression, and deconvolution analysis is performed on the expression matrix of immune cell subtypes. It contains 547 biomarkers that distinguish 25 mouse immune cell phenotypes, including T cells, B cells, plasma cells, and myeloid cell subsets. In this study, the CIBERSORT algorithm was used to analyze the data of the samples to infer the correlation of 25 kinds of immune infiltrating cells with the expression of critical genes in normal samples and disease samples.

### 2.5. Gene Set Enrichment Analysis

The Gene Set Enrichment Analysis (GSEA) analysis uses a predefined gene set to sort the critical genes according to the degree of differential expression in the 2 types of samples, and then checks whether the preset gene set is enriched at the top or bottom of the sorting table. In this study, GSEA was used to compare the differences in signaling pathways between the high and low expression group, and to explore the molecular mechanism of the critical genes of the 2 groups of samples. The number of replacements was set to 1000, and the replacement type was set to phenotype.

### 2.6. Regulatory network analysis of critical genes

In this study, the R package “RcisTarget” was used to predict transcription factors. All calculations performed by RcisTarget are based on motifs. The normalized enrichment score (NES) of a motif depends on the total number of motifs in the database. In addition to motifs annotated by source data, we infer further annotation files based on motif similarity and gene sequence. The first step in estimating the overexpression of each motif across a gene set is to calculate the area under the curve (AUC) for each motif-motif-set pair. This was performed based on the recovery curve calculation of the gene set versus motif ordering. The NES of each motif is calculated from the AUC distribution of all motifs in the gene set. We use mm9-500bp-upstream-10species.mc9nr for the Gene-motif rankings database.

### 2.7. microRNA network construction

The microRNAs (miRNAs) are small non-coding RNAs that have been shown to regulate gene expression by promoting the degradation of mRNAs or inhibiting the translation of mRNAs. Therefore, we further analyzed whether some miRNAs in critical genes regulate the transcription or degradation of some dangerous genes. We obtained miRNAs related to critical genes through the targetscan database, and visualized the miRNA network of genes through cytoscape software.

### 2.8. Connectivity Map drug prediction

The Connectivity Map is a gene expression profiling database based on intervention gene expression developed by the Broad Institute; it is mainly used to reveal the functional relationship between small molecule compounds, genes and disease states. Contains microarray data before and after treatment of 5 cell lines with 1309 small molecule drugs. There are various treatment conditions, including different drugs, different concentrations, different treatment time and so on. In this study, the DEGs of diseases are used to predict the targeted therapeutic drugs of diseases.

### 2.9. Statistical analysis

Statistical analysis was performed using R language (version 4.0). and Prism (GraphPad Prism9, Boston, USA). A significant difference was considered statistically when *P < .05, **P < .01, ***P < .001, or ****P < .0001.

## 3. Results

### 3.1. Determination of DEGs in ALI, and functional enrichment analyses

**Figure 1.**
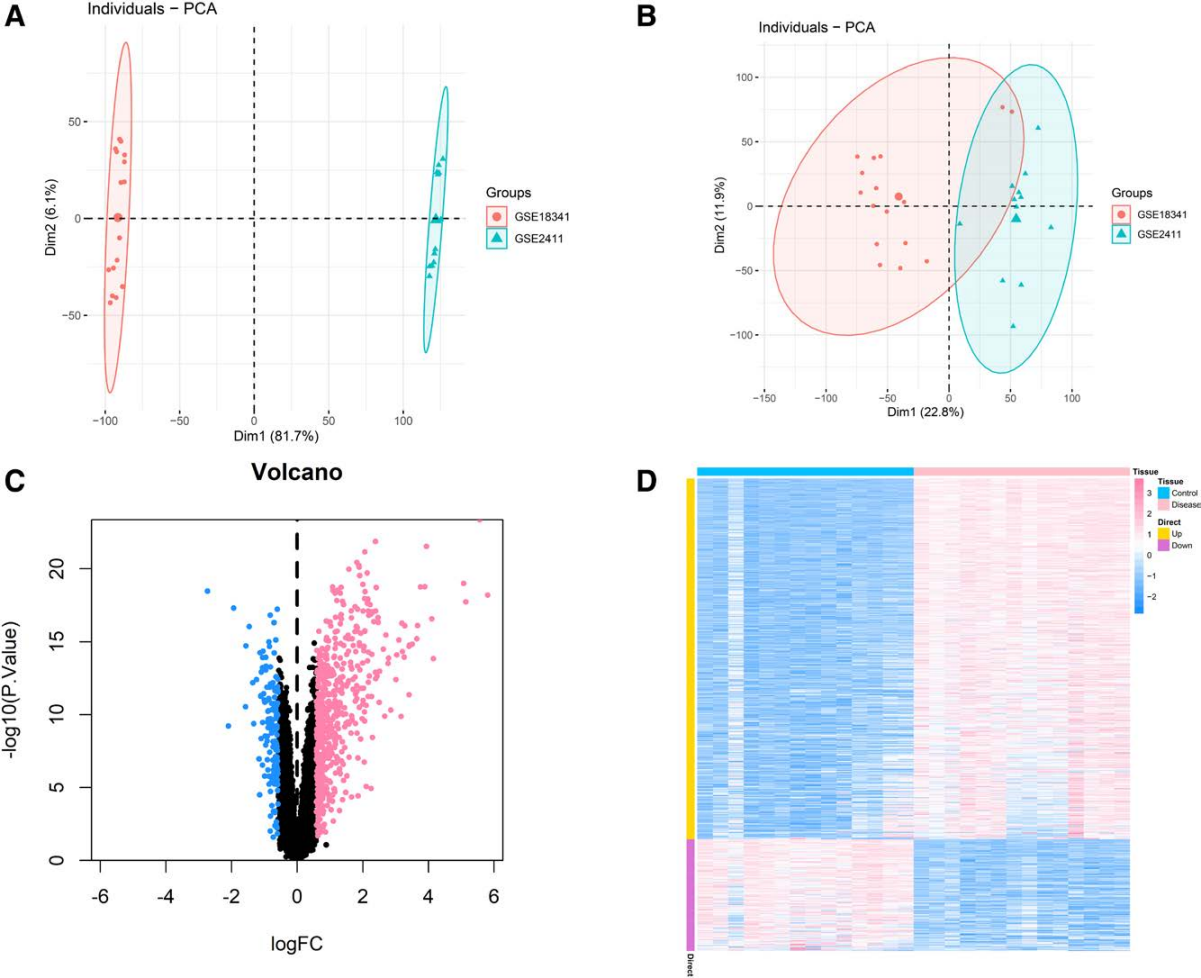
DEGs between ALI and healthy specimens. (A) Before Combat algorithm correction. (B) After Combat algorithm correction. (C) DEGs were selected by volcano plot filtering (adjusted *P* < .05 and | logFC| > 2). Upregulated DEGs were shown in red and downregulated DEGs were shown in blue. (D) DEG expression heatmap between ALI and normal tissues. ALI = acute lung injury, DEGs = differentially expressed genes.

**Figure 2.**
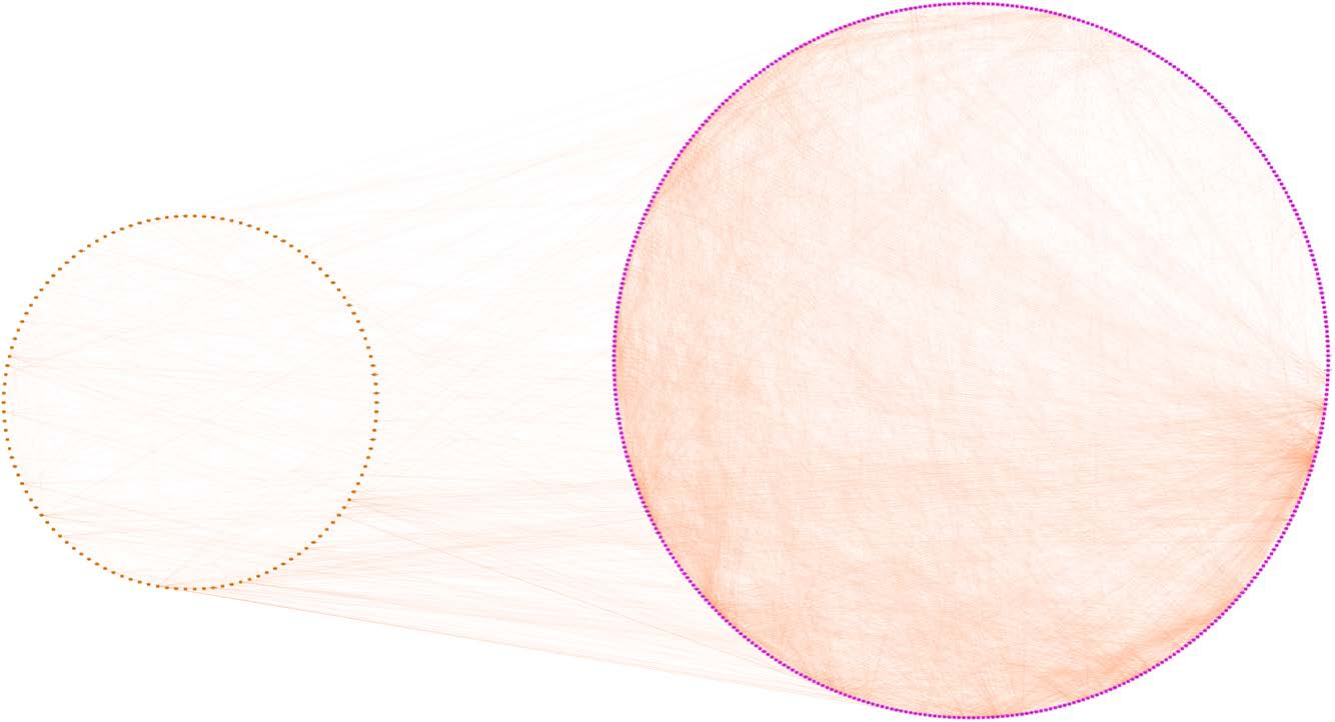
Establishment of PPI network. PPI network of DEGs between ALI and normal controls. ALI = acute lung injury, DEGs = differentially expressed genes.

**Figure 3.**
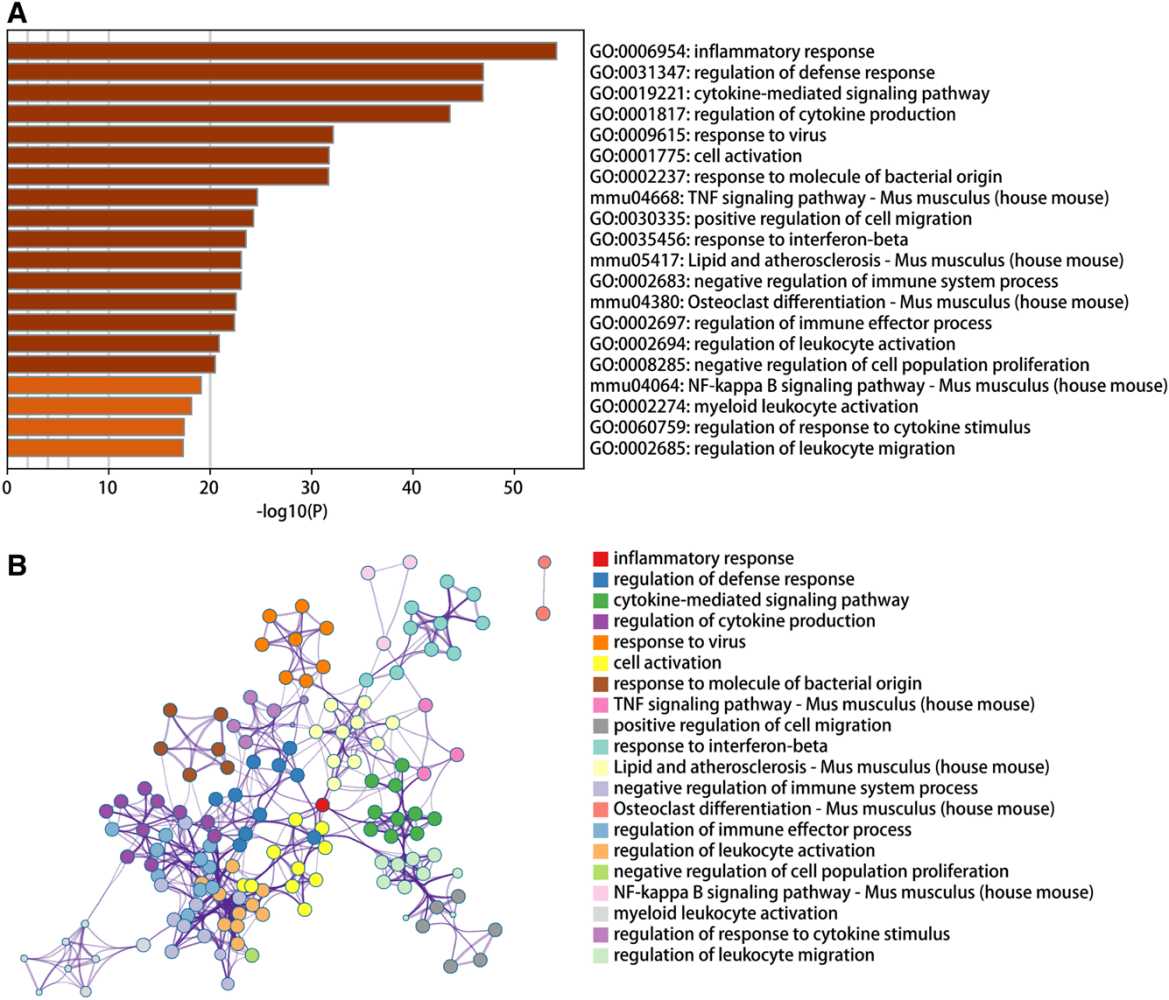
GO and KEGG analyses results of DEGs between ALI and normal controls (A) GO and (B) KEEG pathway. ALI = acute lung injury, DEGs = differentially expressed genes, GO = Gene Ontology, KEGG = Kyoto Encyclopedia of Genes and Genomes.

We downloaded GSE2411 and GSE18341 and ALI-related data sets from the GEO database, and included the expression profile data of 28 groups of samples, including 14 cases in the control group and 14 cases in the disease group. Use the Combat algorithm to correct the chip, and use the PCA graph to show the difference before and after correction. The results show that the batch effect between chips is reduced after correction by the Combat algorithm (Fig. 1A and B). We further used the limma package to calculate the differential genes between the 2 groups of samples. The differential gene screening conditions were: *P*.Value < .05 & |log FC| > 0.585. A total of 690 differential genes were screened, including 527 up-regulated genes and 163 down-regulated genes (Fig. 1C and D). The protein interaction pairs related to differential genes were obtained through STRING online database (http://string-db.org), and visualized through Cytoscape software (Fig. 2). We then conducted pathway analysis on the 690 differential genes, and the results showed that the differential genes were mainly enriched in pathways such as inflammatory response, regulation of defense response, and cytokine-mediated signaling pathway (Fig. 3).

### 3.2. Determination and verification of diagnosis markers

**Figure 4.**
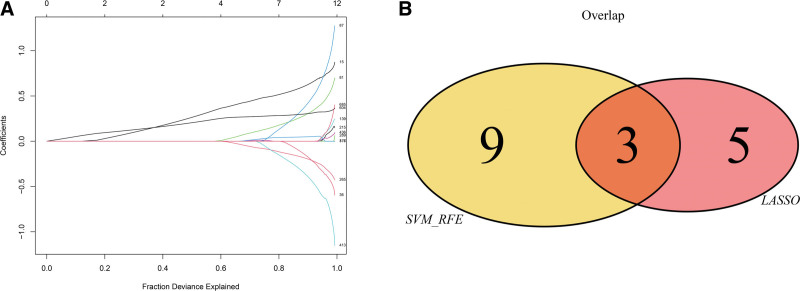
Selection of diagnosis marker candidates for ALI (A) tuning feature screening in the LASSO model. (B) Venn graph displaying 3 diagnosis biomarkers shared by LASSO and SVM-RFE. ALI = acute lung injury, LASSO = least absolute shrinkage and selection operator, SVM-RFE = SVM-recursive feature elimination.

In order to further find out the critical genes affecting ALI, we combined LASSO regression and SVM feature selection algorithm to screen the characteristic genes in ALI among 690 differential genes. The results showed that LASSO regression identified a total of 8 genes as the characteristic genes of ALI (Fig. 4A), on the other hand, we evaluated the characteristic genes in ALI by SVM-recursive feature elimination algorithm. The results showed that by screening the top 12 characteristic genes with the highest accuracy then taking the intersection with the characteristic genes screened out by the LASSO regression algorithm, a total of 3 intersection genes were screened out (Fig. 4B). These 3 genes will be the critical genes of our follow-up research, they are: PDZK1-interacting protein 1 (PDZK1IP1), Cholecystokinin A receptor (CCKAR), and chemokine CXC ligand (CXCL2).

### 3.3. Critical genes are Related to Immunocyte Infiltration Levels.

**Figure 5.**
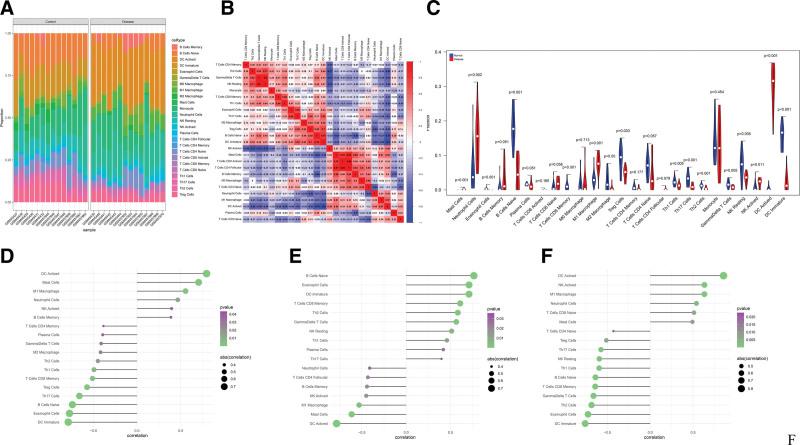
Immune infiltration levels. (A and B) The percentage of the 25 immunocytes identified via the CIBERSORT arithmetic. (C) The diversities in the architecture of immunocytes between ALI and healthy specimens. Correlation between (D) PDZK1IP1, (E) CCKAR, (F) CXCL2 and infiltrating immune cells in ALI and normal samples. ALI = acute lung injury.

**Figure 6.**
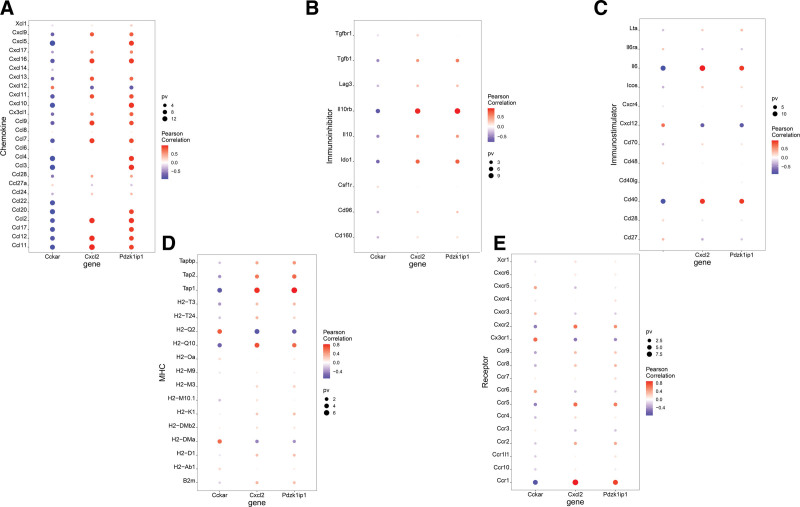
Establishment of Immune association. Correlations between critical genes and different immune factors (A) Chemokine. (B) Immunoinhibitor. (C) Immunostimulator. (D) MHC. (E) Receptor.

The immune microenvironment is mainly composed of immune-related fibroblasts, immune cells, extracellular matrix, various growth factors, inflammatory factors and special physical and chemical characteristics. The immune microenvironment affects the diagnosis, survival outcome and clinical treatment sensitivity of diseases significantly. We explored the mechanism by which critical genes affect the progression of ALI by analyzing the relationship between critical genes and immune infiltration in the disease dataset. The immune cell content of each sample is shown in the figure (Fig. 5A). There were multiple significant correlation pairs between the levels of immune infiltration (Fig. 5B). And compared with normal samples, the levels of Mast Cells, Neutrophil Cells, M1 Macrophage, and dendritic cell (DC) Actived in disease group samples were significantly higher (Fig. 5C). Then we explored the relationship between critical genes and immune cells, and found that several critical genes are highly correlated with immune cells, PDZK1IP1 is positively correlated with DC Actived, Mast Cells, etc., and negatively correlated with DC Immature, Eosinophil Cells, etc. (Fig. 5D); CCKAR is positively correlated with B Cells Naive, Eosinophil Cells, etc., and negatively correlated with DC Actived, Mast Cells (Fig. 5E); CXCL2 is positively correlated with DC Actived, NK.Actived, etc., and negatively correlated with DC Immature, Eosinophil Cells, etc. (Fig. 5F). We also obtained the correlation between these critical genes and different immune factors, including immune modulators, chemokines, and cell receptors, etc. from the TISIDB database (Fig. 6). These analyzes confirmed that these critical genes are closely related to the level of immune cell infiltration and play important role in the immune microenvironment.

### 3.4. miRNA network Prediction and GSEA analysis.

**Figure 7.**
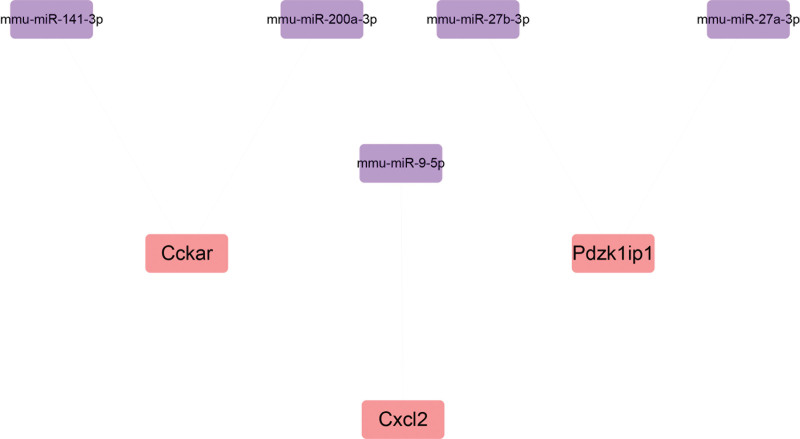
miRNA network prediction. miRNA = microRNA.

**Figure 8.**
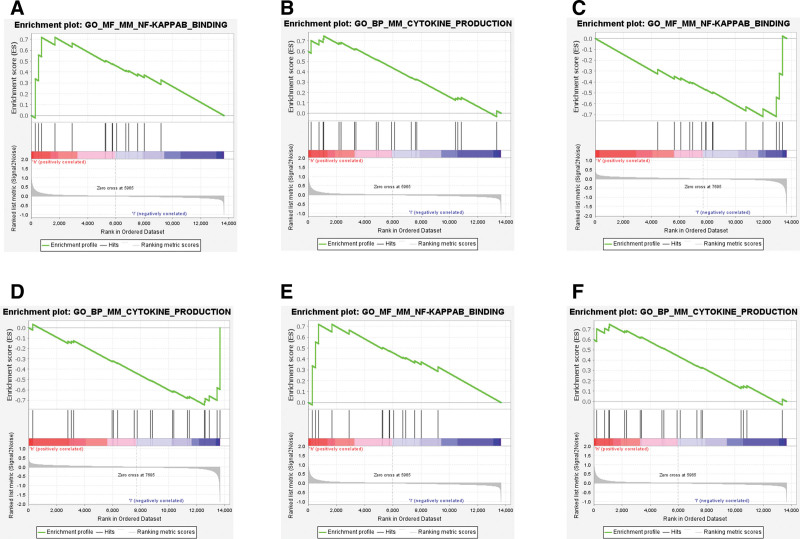
GSEA analysis revealed that the critical genes. (A and B) PDZK1IP1, (C and D) CCKAR, (E and F) CXCL2 were mainly enriched in NF-KAPPAB_BINDING, CYTOKINE_PRODUCTION related pathways. GSEA = Gene Set Enrichment Analysis.

We also performed reverse prediction on the 3 critical genes through the targetscan database, and obtained 5 miRNAs and 5 pairs of mRNA-miRNA relationship pairs, which were visualized by cytoscape (Fig. 7). Then, we studied the specific signaling pathways involved in the 3 critical genes, and explored the underlying molecular mechanisms by which critical genes affect disease progression. The results of The GSEA analysis showed that highly expressed PDZK1IP1, CCKAR, and CXCL2 was mainly enriched in NF-KAPPAB_BINDING; CYTOKINE_PRODUCTION and other signaling pathways (Fig. 8A–F).

### 3.5. Motif enrichment analysis

**Figure 9.**
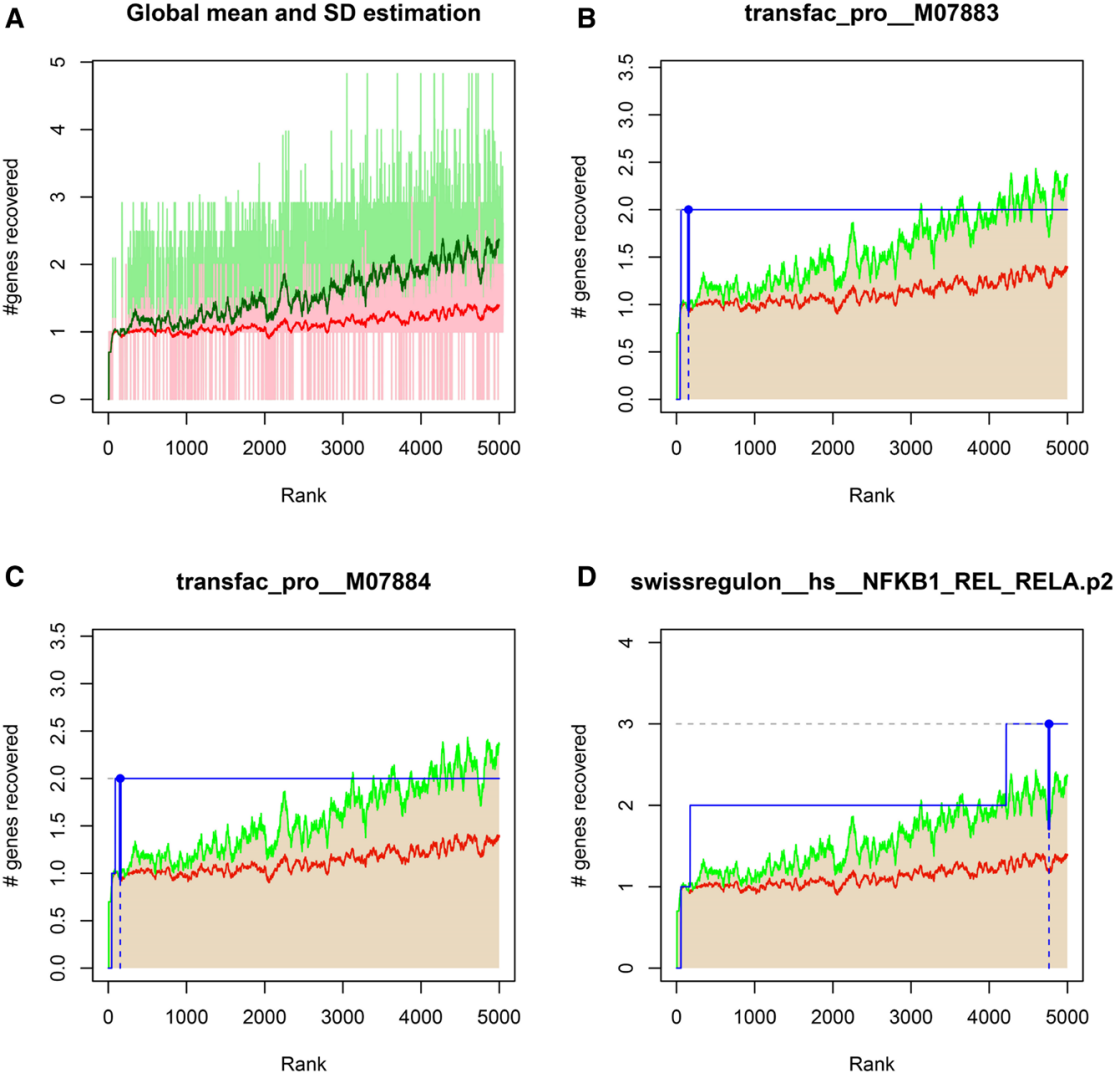
Motif enrichment analysis on these transcription factors by cumulative recovery curves. (A) Transcription factors using cumulative recovery curves, (B) transfac_pro__M07883, (C) transfac_pro__M07884, (D) Swissregulon_hs_NKKB1_REL_RELA.p2.

We used 3 critical genes for the gene set in this analysis and found that they are regulated by multiple transcription factors. Therefore, enrichment analysis was performed for these transcription factors using cumulative recovery curves (Fig. 9A). The analysis results showed that the motif with the highest AUC was transfac_pro__M07883, and 2 critical genes were enriched in this motif, namely: CXCL2 and PDZK1IP1, with a NES of 8.7. In addition, we display all enriched motifs and corresponding transcription factors for critical genes (Fig. 9B–D).

### 3.6. Correlation analysis between critical genes and disease gene

**Figure 10.**
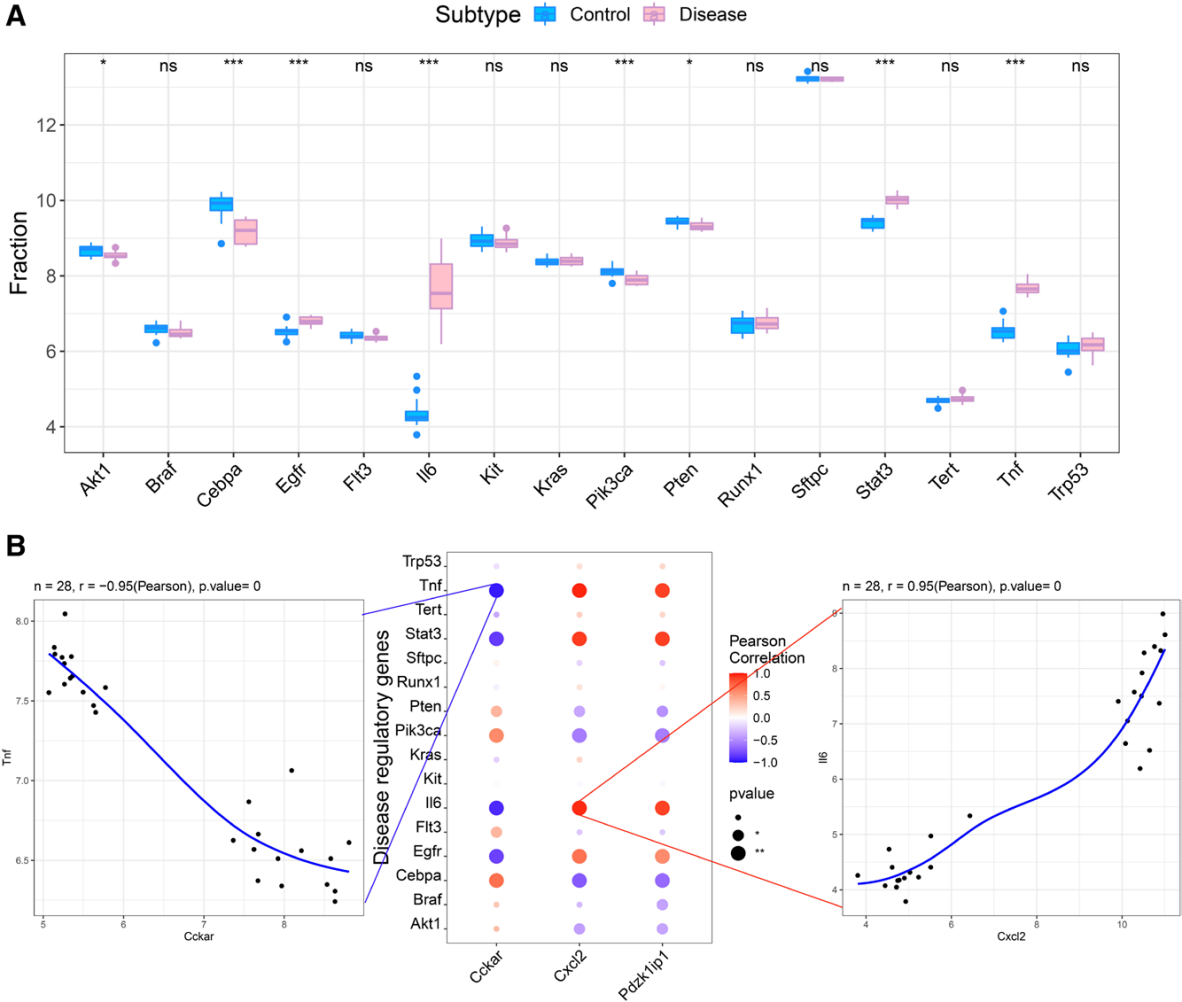
Correlation analysis. (A) Expression differences of ALI pathogenic genes among groups. (B) Correlation analysis between critical genes and disease gene. ALI = acute lung injury.

The disease genes related to ALI were obtained through the GeneCards database (https://www.genecards.org/). Analyzing the differences in the expression of ALI pathogenic genes between groups, it was found that the expressions of Akt1, Cebpa, Egfr, IL6, Pik3ca, Pten, Stat3, and TNF genes were different in the samples of the 2 groups (Fig. 10A). Then, we performed a correlation analysis on the critical genes and regulatory genes in ALI, and found that the expression levels of these 3 genes were significantly correlated with the expression levels of multiple disease-related genes, and CXCL2 and IL-6 were significantly positively correlated (*r* = 0.95), CCKAR and TNF were significantly negatively correlated (*r* = −0.95) (Fig. 10B).

## 4. Discussion

ARDS/ALI is a clinical syndrome characterized by refractory hypoxemia caused by multiple pathogenic factors. The disease is serious and there is no effective treatment, so it is necessary to find an effective target.

In this study, we aimed to identify novel diagnostic genes for ALI based on bioinformatics and machine learning. We analyzed 2 GEO datasets (GSE2411 and GSE18341) to determine DEGs between ALI and healthy specimens. A total of 690 differential genes were screened out. We then conducted pathway analysis on these 690 differential genes. The results showed that the differential genes were mainly involved in pathways such as inflammatory response, regulation of defense response, and cytokine-mediated signaling pathway.

A total of 690 differential genes were screened out. We further conducted pathway analysis on these 690 differential genes. The results showed that the differential genes were mainly involved in pathways such as inflammatory response, regulation of defense response, and cytokine-mediated signaling pathway. It is well established that these pathways play key role in ALI: Cellular responses in host defense response in ALI are regulated by a complex interaction among cytokines leading to persistent inflammation, and form a cytokine inflammatory storm through cytokine cascade amplification in ALI.^[10–12]^ thereby exacerbating the progression of ALI/ARDS. The results of the analysis indicated that these DEGs were actively involved in the immune inflammatory response and might play a role in the development of ALI. To screen potential diagnostic biomarkers for ALI, we performed machine learning algorithms by the use of the above 690 DEGs, and 3 critical genes were identified, namely PDZK1IP1, CCKAR, and CXCL2.

Recently, mounting researches have revealed that immunocyte infiltration was vital for the onset and developmental process of ALI. Neutrophils, DCs, and macrophages are associated with multiple immunological processes and tissue injury by initiating inflammation during ALI.^[13]^ Cheng and his group revealed that ALI presented with macrophage infiltration.^[13,14]^ ALI have been discovered to display an evident feature of neutrophils infiltration. these infiltrated neutrophils block pulmonary microcirculation due to their prolonged entrapment in the capillaries which further aggravates ALI.^[15,16]^

CXCL2, also known as macrophage inflammatory protein-2,^[17]^ is a CXC chemokine that plays an important role in the process of inflammation injury repair and has important physiological functions such as participating in the reorganization of the cytoskeleton, cell migration, adhesion and immune responses.^[18]^ CXCL2 affects neutrophil recruitment and activation by binding to its specific receptors, CXCR1 and CXCR2.^[19]^ In mice, by inhibiting the expression of CXCL2, the researchers found that the recruitment of neutrophils in the lung was reduced, and the damage of ALI was attenuated.^[20,21]^ On the contrary, local inflammation was aggravated when the expression of CXCL2 was elevated. Furthermore, our study shows that the expression of CXCL2 is highly correlated with IL-6,^[22]^ which play critical role in the inflammatory response. This provides a new idea for the intervention treatment of ALI.^[23]^

PDZK1IP1 (MAP17) is a small, 17 kDa non-glycosylated membrane protein located in the plasma membrane and Golgi apparatus. It is overexpressed in a wide variety of human carcinomas and has been proposed as a predictive biomarker for acute coronary syndrome, reactive oxygen species and treatments in cervical tumors or laryngeal carcinoma.^[24,25]^ PDZK1IP1 expression directly regulates NFAT2 and IL-6 activation, inducing the differentiation of monocytes to dendritic cells and suggesting a causal role of PDZK1IP1 in inflammation.^[26]^ Furthermore, the ectopic expression of PDZK1IP1 increases glucose and mannose uptake, generating an increase in reactive oxygen species levels that can exacerbate tissue damage and pulmonary edema and thus aggravate ALI.^[27,28]^

CCKAR is a G proteincoupled receptor that can be activated by cholecystokinin.^[29]^ It has been found to be associated with tumor suppression and plays an important role in the physiopathological process of gallbladder cancer and esophageal cancer. As a key factor in the P38 pathway, CCKAR inhibits the release of inflammatory factors and thus inhibits the process of inflammation.^[30,31]^ In addition, CCKAR has also been demonstrated to be beneficial in reducing TNF absorption and attenuating gut mediator-induced lung damage during endotoxemia.^[32]^ This is consistent with the result of our study that CCAKR is negatively correlated with TNF which is a key factor of ALI,^[33]^ however, its mechanism remains to be studied.

Hence, from the perspective of the immune system, evaluating the infiltration of immunocytes and identifying the diversities in the constituents of infiltrating immunocytes were imperative for revealing the molecule level causal link beneath ALI and designing novel immune therapy targets. In this study, we found that there were significant differences in Mast Cells, Neutrophil Cells, M1 Macrophage, DC Actived, Eosinophil Cells, B Cells Naive, Mast Cells, and DC Immature between the control group and the ALI group. Neutrophils, Mast Cells, and M1 macrophages are inextricably linked to the progression of ALI. However, its specific role and mechanism need to be further studied.^[16,34,35]^

We predicted 5 miRNAs that may be related to critical genes through bioinformatics, which provided a new direction for ALI treatment. We also identified a TF regulatory network associated with critical genes and found that cis transfac_pro_M07883 was the most significantly enriched motif, indicating a potential binding site for TFs. In addition, it is also vital to understand the molecular mechanism of gene pathogenesis by exploring signaling pathways.^[12]^ Studies have shown that signaling pathways such as NF-kB and cytokine production are important mediators of ALI. Our study confirmed that PDZK1IP1, CCKAR, and CXCL2 were enriched in these pathways, and showed a trend of up-regulation or down-regulation. We speculate that these genes contribute to ALI through these pathways, However, these predictions require further studies to be validated.

Therefore, Based on the text you provided, it seems that PDZK1IP1, CCKAR, CXCL2 are associated with the occurrence and progress of ALI via regulating several immune cells. However, there are still some limitations to be acknowledged. First, data herein can merely support the correlative analyses between ALI and immunocytes, rather than revealing the causality. To address the limitations in this study, research using knockout gene mice for each DEGs is indispensable and in urgent need. It will facilitate us to get better acquainted with its role of LPS in aggravating ALI/ARDS. Second, survival analysis on adequate clinical samples is required.

## 5. Conclusion

In summary, our study provides an integrated bioinformatics analysis of DEGs of ALI. The research shows that PDZK1IP1, CCKAR and CXCL2 are key DEGs in ALI compared to healthy specimens. These key DEGs have been described for the first time in exploring DEGs in ALI study. The findings in the present research may offer enlightenment for revealing the potential molecule level causal links of ALI. They also serve as new molecular biomarkers for exploring new diagnosis and prognosis for ALI. At the same time, this study also has certain limitations. The regulatory functions of these key DEGs in ALI have not been fully known. Therefore, to verify the current findings, it is necessary to perform further studies of animal and clinical research.

## Author contributions

**Conceptualization:** Hekun Jing, Daoxin Wang.

**Data curation:** Daoxin Wang.

**Funding acquisition:** Daoxin Wang.

**Investigation:** Hekun Jing, Xiaorui Chen.

**Methodology:** Hekun Jing, Xiaorui Chen.

**Project administration:** Hekun Jing.

**Resources:** Hekun Jing.

**Software:** Hekun Jing.

**Supervision:** Hekun Jing, Xiaorui Chen.

**Validation:** Hekun Jing, Xiaorui Chen.

**Visualization:** Hekun Jing.

**Writing – original draft:** Hekun Jing, Xiaorui Chen.

**Writing – review & editing:** Daoxin Wang.
